# Contactless cardiac gating at 0.55T using high‐amplitude pilot tone with interference cancellation (HAPTIC)

**DOI:** 10.1002/mrm.30528

**Published:** 2025-04-14

**Authors:** Bilal Tasdelen, Ecrin Yagiz, Baran R. Cinbis, Ye Tian, Krishna S. Nayak

**Affiliations:** ^1^ Ming Hsieh Department of Electrical and Computer Engineering University of Southern California Los Angeles California USA

**Keywords:** 0.55T MRI, cardiac phase, interference cancellation, pilot tone, respiratory phase

## Abstract

**Purpose:**

To enable contactless cardiac gating at 0.55T using pilot tone (PT). Current PT methods are unable to extract weak motions, including cardiac motion, at lower B_0_ field strengths (<1.5T).

**Methods:**

We utilize high‐amplitude pilot tone with interference cancellation, termed HAPTIC. The use of high amplitude PT improves sensitivity to cardiac motion, but introduces noise leakage into the imaging bandwidth. This leakage is removed using External Dynamic InTerference Estimation and Removal (EDITER) interference cancellation. HAPTIC performance at 0.55T is evaluated in healthy volunteers and patients with cardiac arrhythmia, over a 100‐fold range in PT amplitude. Contactless HAPTIC gating performance is compared against conventional electrocardiogram (ECG). Noise enhancement due to HAPTIC is evaluated using noise‐only scans acquired with varying PT amplitude levels.

**Results:**

We demonstrate robust extraction of cardiac PT signals at 0.55T, with cardiac gating (ECG vs. HAPTIC) jitter <9 ms, and noise enhancement ˜12%–35%. We demonstrate the ability to track cardiac and respiratory phase during real‐time MRI and demonstrate reliable separation of cardiac and respiratory phases for retrospective binning using HAPTIC. Furthermore, we demonstrate that HAPTIC provides accurate cardiac gating in the challenging case of arrhythmia to showcase initial feasibility.

**Conclusion:**

HAPTIC enables contactless cardiac gating at 0.55T, which has not previously been demonstrated with any PT variant. This could simplify clinical workflow and could serve as a solution for mid‐ and low‐field MRI scanners that do not include built‐in physiological monitoring.

## 1 INTRODUCTION

Pilot tone (PT) is a novel contact‐less method for capturing body motion remotely, for example, head, cardiac, and/or respiratory motion.^1^, ^2^, ^3^ PT can serve as a replacement for electrocardiogram (ECG) or plethysmograph (PG) cardiac monitoring, and for bellow or imaging navigator respiratory tracking. This simplifies patient preparation, improves patient comfort, and simplifies scan planning. PT also makes it possible to perform cardiac gating on scanners that do not come with an integrated ECG or PG.^4^

One known caveat is that the PT navigator quality is highly dependent on the B_0_ field strength. The PT signal modulation due to motion is directly proportional to the resonance frequency and, therefore the B_0_ field strength.^5^ This is less of an issue for bulk motion, such as respiratory motion; however, it makes the extraction of weaker motion, for example, cardiac motion, extremely challenging on mid‐ and low‐field systems.^6^ To date, there has been no successful demonstration of PT cardiac gating at <1.5T.

In this work, we overcome this limitation by using high‐amplitude pilot tone with interference cancellation (HAPTIC) to obtain cardiac and respiratory signals at 0.55T. We demonstrate this enables the extraction of reliable cardiac and respiratory phases at 0.55T without substantially compromising image quality. This approach incurs an added cost of enabling additional receiver coils distant to the imaging volume for interference measurement (a.k.a. sniffer coils) and a modest noise penalty (<12% increase). We evaluate feasibility in a small cohort of two healthy volunteers and four arrhythmia patients.

## 2 METHODS

### 2.1 HAPTIC

Motion estimation in PT comes from the modulation of the PT waveform, defined as the ratio between the change of the waveform and the carrier amplitude. This modulation is dependent on the carrier frequency, receiver coil placement, and the nature of the motion. One way to increase detection sensitivity is to increase the carrier amplitude. Conventionally, the carrier amplitude is kept small in order to limit potential effects on the image quality. Image quality can degrade due to PT noise leakage into the imaging bandwidth or by saturating the receiver. The central idea in HAPTIC is to use high carrier amplitudes to improve motion sensitivity and then mitigate the PT signal leakage using interference cancellation. There is still a possibility of saturating the receiver resulting in quantization noise, which we evaluate experimentally.

**FIGURE 1:**
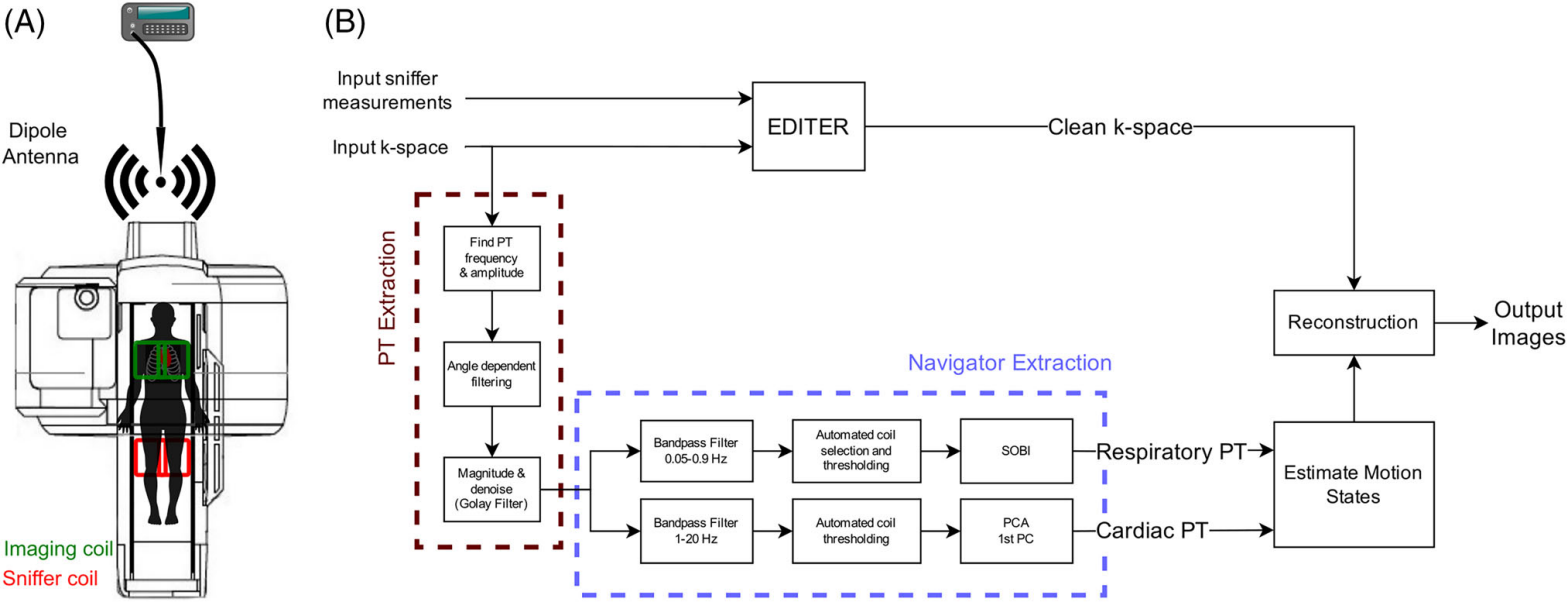
Pilot tone (PT) setup and image reconstruction pipeline. (A) Sketch of the placement of signal generator and sniffer coils for the pilot tone experiment. Unlike the vendor‐integrated or other home‐built PT hardware, our PT antenna is away from the bore (˜1 m). Furthermore, instead of a non‐resonant antenna, we utilize a resonant dipole antenna for PT transmission. (B) Signal processing and image reconstruction pipeline, describing the PT extraction and interference cancellation.

We used an in‐house PT setup comprised of a general‐purpose signal generator (AFG3252, Tektronix) and a resonant dipole antenna. Figure 1 illustrates the experiment setup. The signal generator antenna is positioned 1 m away from the back of the scanner. The PT frequency is set approximately 400 kHz away from the imaging center frequency. As the signal generator is not synchronized to the scanner, we discard the phase of the carrier and only utilize the amplitude modulations in this study.

First, frequency, then amplitude of the PT carrier per coil and per k‐space line are estimated. The amplitude per TR is used as the raw PT waveform. The amplitude per TR is used as the raw PT waveform. The samples of the waveform are reordered according to the starting angle of each spiral arm. The reordered samples are processed with a low‐pass filter along the angle dimension to remove trajectory‐dependent deviations (e.g., eddy‐currents)^7^ followed by a Savitzky‐Golay^8^ filter for denoising. Denoised waveforms are then band‐pass filtered in the respiratory (0.05–0.9 Hz) and cardiac (1–20 Hz) ranges separately. Cross‐correlation matrices for respiratory‐filtered waveforms are calculated, and the channel with the highest total correlation to the other channels is selected as the initial channel. Any other channels with cross‐correlation higher than 0.9 to the initial channel are accepted for respiratory waveform extraction. The number of accepted channels is dataset dependent and varied between 9 and 13. The respiratory waveform is estimated through Second‐Order Blind Identification (SOBI)^9^ using the accepted channels only.

One channel with strong cardiac modulation is manually selected according to its physical proximity to the heart. This channel is used to identify other channels with cardiac modulations using the cross‐correlation method, similar to the respiratory extraction. Different from the respiratory extraction, the correlation threshold is decreased iteratively from 0.9 down to 0.5 with 0.05 steps until more than two cardiac channels are accepted. The number of selected cardiac channels varied between two and six. Principal component analysis (PCA) is applied to the accepted channels, and the first principal component is used as the cardiac waveform.

At high amplitudes, PT is expected to produce interference in the MRI imaging bandwidth. This interference is removed using the External Dynamic InTerference Estimation and Removal (EDITER) algorithm.^10^ EDITER requires additional coils that measure interference, which we will call *sniffer* coils, on top of the usual coils we use for the signal reception, which we will call *imaging* coils. We used the three most distant elements from the 18‐channel table‐integrated spine array as the sniffer coils. This was chosen for convenience (no additional coils needed to be placed) and to ensure sniffer coils measure minimal signal from the imaging volume. The use of closer sniffer coils that are sensitive to the imaging volume could lead to partial removal of the desired signal along with the PT interference.

As a baseline for comparison, we also implemented the model‐based subtraction method described in Solomon et al.^6^ A sinusoidal PT signal model is assumed, and its frequency, amplitude, and phase are estimated from the acquired data. Then, the estimated PT signal is subtracted from every k‐space readout.

### 2.2 Experimental methods

Experiments were performed using a whole‐body 0.55T system (prototype MAGNETOM Aera, Siemens Healthineers, Forchheim, Germany) with gradients capable of operating at 45 mT/m amplitude and 200 T/m/s slew rate.^11^ The body coil was used for RF transmission, and the center frequency was approximately 23.6 MHz. We used a 6‐channel body array (anterior) and 8 elements from the table‐integrated 18‐channel spine array (posterior) as imaging coils. ECG was simultaneously recorded using a 4‐lead built‐in ECG device (Siemens Healthineers, Forchheim, Germany). Imaging was performed on two healthy adult volunteers (one M/one F, ages 29) and four patients with cardiac arrhythmia (two M/two F, ages 58–75). All volunteers were imaged under protocols approved by our Institutional Review Board, after providing written informed consent.

An in‐house real‐time golden angle spiral balanced SSFP (bSSFP) sequence^12^, ^13^, ^14^ was used for image acquisition, developed in Pulseq.^15^ Imaging parameters were: TR = 5.32 ms, flip angle = 100°, TE = 0.83 ms, in‐plane spatial resolution = 2 × 2 mm^2^, slice thickness = 8 mm. Scan plane was mid short‐axis. Because we estimate scalar amplitude per readout, the sampling period of PT navigators are dictated by the TR. For healthy volunteers, 90 s of real‐time images were acquired, followed by 30 s of noise‐only scan with the same acquisition parameters for each PT setting as described below. Ninety seconds of real‐time images were acquired for arrhythmia subjects only for a single, predetermined setting of 0.4 V.

### 2.3 Image reconstruction and evaluation

PT estimation and image reconstruction were performed offline. Image reconstruction was performed with Berkeley Advanced Reconstruction Toolbox (BART)^16^ and using the gradient impulse response function^17^ corrected spiral trajectory. View‐sharing reconstruction is performed for noise analysis and demonstration of the high‐amplitude PT artifacts. It used a temporal resolution of 17 arms/frame (93 ms) with a temporal footprint of 55 arms/frame (300 ms). Temporal finite difference (TFD) constrained reconstruction is used to reconstruct real‐time movies of arrhythmia patients, and it uses 7 arms/frame (38 ms).^18^ Spiral arms were binned into 12 respiratory and 20 cardiac phases for dual temporally constrained reconstruction, similar to eXtra‐Dimensional Golden‐angle RAdial Sparse Parallel (XD‐GRASP),^19^ which is used to demonstrate cardiac and respiratory signal from HAPTIC is reliable enough to separate the motion successfully.

The HAPTIC estimated cardiac waveform was compared against simultaneously acquired ECG measurements. Each HAPTIC cardiac peak is defined as follows: The first derivative of the HAPTIC cardiac waveform is computed and then normalized such that the 10th percentile is 0 and the 98th percentile is 1. Each cardiac peak is the local maximum of this waveform within a 0.375‐second window, corresponding to a 160 bpm maximum detectable heart rate. Each peak must have a prominence, defined as the amplitude distance between the peak and its lowest contour line (the higher base on either side of the peak), greater than 0.5. The precise implementation is shared (see Data Availability Statement). ECG‐PT jitter is used as the performance metric and is calculated by measuring the time difference between each HAPTIC cardiac peak and its corresponding ECG R‐wave peak. Jitter is defined as the SD of the PT minus ECG time difference. The number of HAPTIC peaks between any two ECG R‐waves is counted. The first HAPTIC peak found is matched with the preceding R‐wave. If there is more than one HAPTIC peak within the search window, these additional peaks are considered false positives, and if no HAPTIC peak is detected, it is considered a false negative.

We anticipate the possibility of noise increase due to residual PT interference that is not fully canceled. We therefore measure and report a noise ratio. This is estimated from noise‐only scans, and is the ratio between the noise SD of the image with HAPTIC on divided by the noise SD of the reference image with no PT transmission and HAPTIC off. Noise SD was calculated from a circular 16 cm diameter region‐of‐interest at the center of the FOV in the noise‐only images.

PT amplitude sweep experiments were performed on a healthy volunteer to determine jitter and noise amplification trade‐off with respect to PT amplitude. The reported amplitude is the voltage set for the signal generator output. Ten amplitudes (0.01, 0.02, 0.04, 0.06, 0.08, 0.1, 0.2, 0.4, 0.6, 0.8, and 1 V) were acquired. Both jitter and noise increase for baseline and EDITER applied images were plotted against the PT amplitude. Four representative baselines and EDITER processed images selected across the voltage range were qualitatively compared.

## 3 RESULTS

**FIGURE 2:**
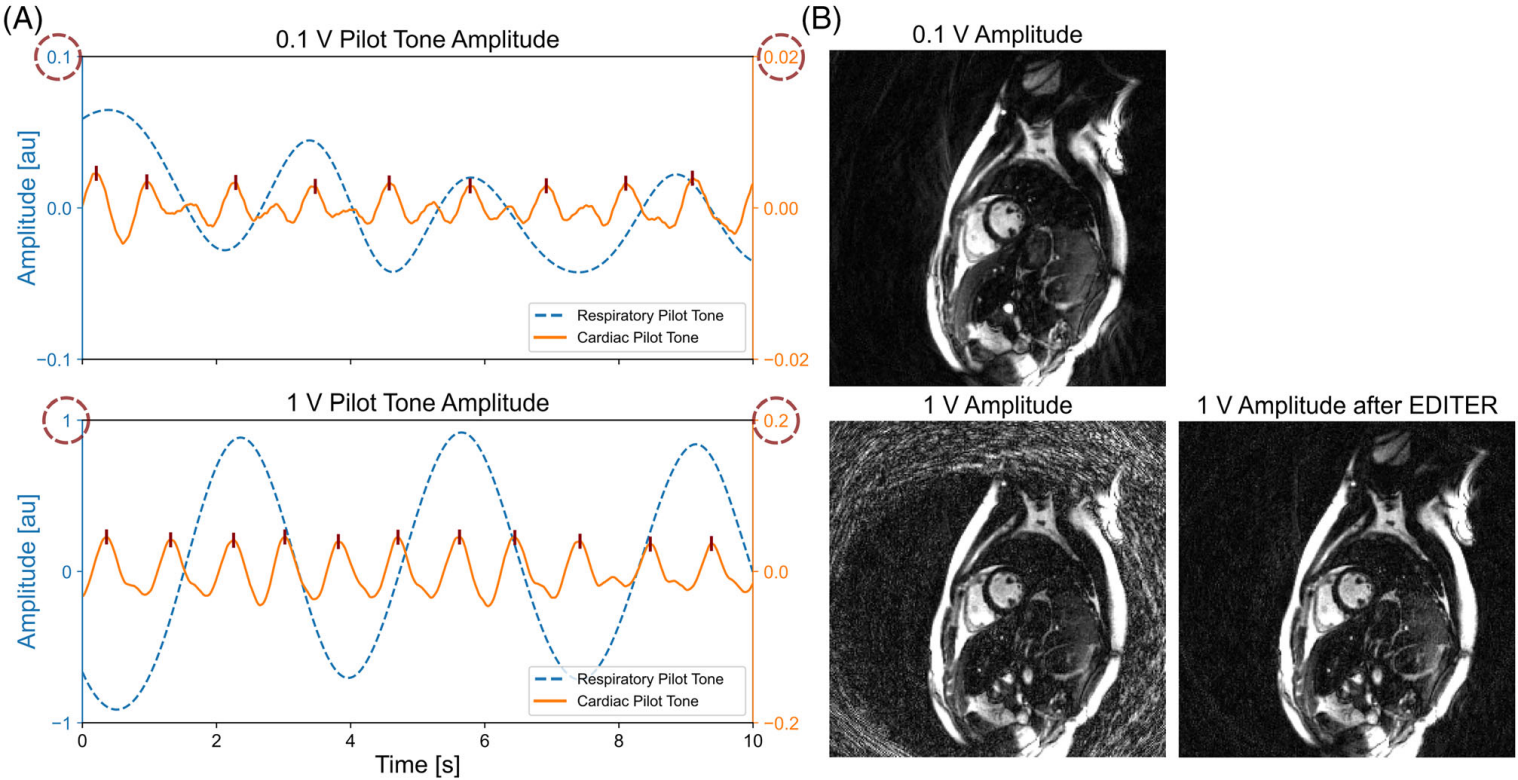
Illustration of the effect of high amplitude pilot tone (PT) and its mitigation. (A) The plots show the extracted pilot tone navigators with lower amplitude pilot tone (0.1 V, top row) and with amplitude 10 times higher than the reference (1 V, bottom row). Note the 10× scale difference of y‐axes. Trigger points are marked as red ticks. Increased PT amplitude results in a proportional increase in the navigator modulations. Higher amplitudes provide higher fidelity navigators, especially more pronounced with cardiac navigator. Cardiac navigator with higher PT amplitude has more pronounced peaks and better signal SNR, which makes cardiac gating more reliable. (B) The images show still frames from view‐sharing reconstructed movies with reference amplitude (0.1 V, top row) and 1 V PT amplitude (bottom row). The bottom row frame also shows the frame for 1 V PT amplitude after interference removal with external dynamic interference estimation and removal (EDITER). The temporal footprint of view‐sharing was 300 ms. Higher amplitude PT induces severe artifacts, more pronounced towards the edges of the FOV. EDITER was able to greatly reduce artifacts from PT.

Increasing the PT amplitude improved navigator fidelity, which was especially noticeable for cardiac navigators, as illustrated in Figure 2A. However, this also introduced artifacts due to residual PT carrier and noise leaking into the imaging bandwidth. Figure 2B shows these artifacts and demonstrates EDITER's effectiveness in removing the artifacts. Artifacts are more severe towards the edge of the FOV, as expected because the PT carrier is beyond the imaging bandwidth. In all cases that were tested, EDITER interference cancellation reduced the severity of the artifacts and improved perceived signal‐to‐noise ratio (SNR).

**FIGURE 3:**
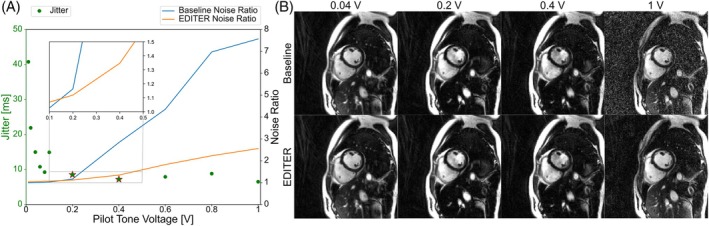
Tradeoff between pilot tone (PT) amplitude, cardiac gating jitter, and noise ratio. (A) Pilot‐tone amplitude versus jitter plot. Jitter decreases and noise ratio increases as the amplitude increases, which presents a trade‐off. A setting of 0.2 V provides 8.5 ms jitter and 12% noise increase. A setting of 0.4 V provides 7.2 ms jitter and 34.7% noise increase. (B) Still frames from view‐sharing reconstructions showing the artifact levels for the selected four amplitude PTs for external dynamic interference estimation and removal (EDITER) correction and model subtraction (baseline). For low‐amplitude, model subtraction provides sufficient image quality; however, jitter is high. For 0.2 and 0.4 V, EDITER subtraction provides lower noise compared to baseline. For high amplitude of 1 V, even though EDITER provides better image quality, loss of SNR is apparent. *Note*: The images show slightly different SSFP (bSSFP) banding artifact in the liver and chest wall. This is due to a small center frequency drift during this long experiment that tested many PT amplitudes. In all settings, the bands did not interfere with the visualization of the heart.

When the PT amplitude was increased, we observed a trade‐off between reduced jitter (advantage) and increased image noise (disadvantage). The PT amplitude sweep experiment shown in Figure 3A demonstrates that, as the amplitude increases, the jitter reduces until 0.4 V, with negligible change beyond. As expected, the noise ratio increased monotonically with the PT amplitude. Considering these two plots, the desired PT amplitude can be chosen to minimize SNR loss and the jitter simultaneously. We highlighted 0.2 and 0.4 Volts, as they provide low jitter (8.5 and 7.2 ms) and acceptable noise increase (12% and 34.7%) and were used in the remainder of this work. Figure 3B compares the image quality for four PT amplitudes across the sweep between the baseline (without) and with EDITER cancellation. At low amplitudes, model subtraction provided sufficient image quality; however, the jitter was high. For 0.2 V, EDITER subtraction provided lower noise compared to baseline. Even though EDITER delivered better image quality at high amplitudes, the SNR loss is significant.

**FIGURE 4:**
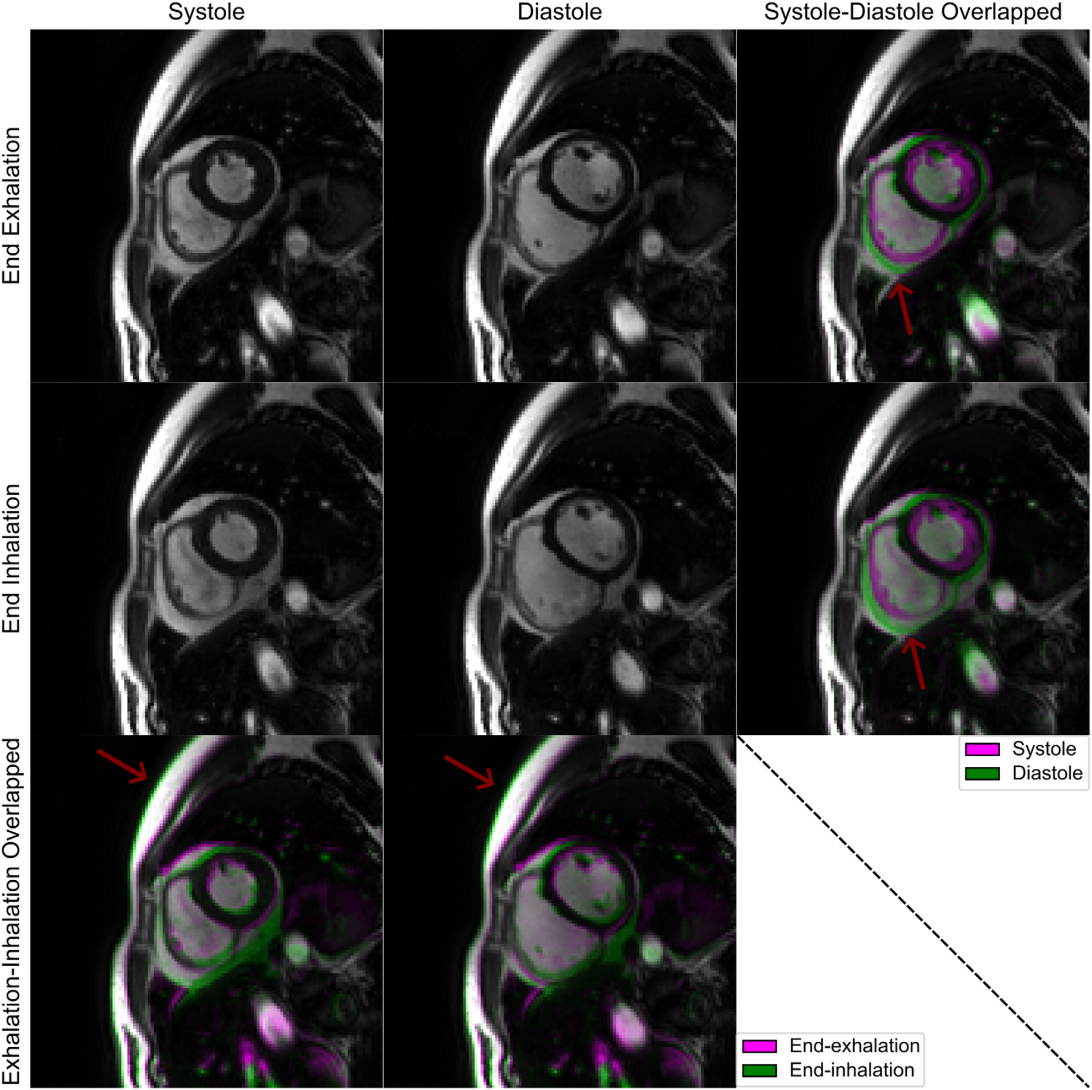
Extreme states of cardiac and respiratory phases from constrained reconstruction using pilot tone (PT) as the sole motion navigator. Columns show systole and diastole phases from left to right, and rows show end‐exhalation and‐inhalation from top to bottom. Overlapped images show the differences between systole and diastole images at the rightmost column, where systole is encoded as magenta and diastole is green, and differences between end exhalation and inhalation at the last row, where end inhalation is encoded as green and end exhalation is magenta. Red arrows point out to important features where the difference can be appreciated. Video S1 shows the cardiac and respiratory states.

Next, we evaluated retrospective gating using HAPTIC to resolve the cardiac and respiratory states. Extreme motion states of dual temporally constrained reconstructed images are shown in Figure 4. The combination of end‐inhalation/exhalation and systole/diastole phases are shown, and the difference images are color‐coded to highlight the motion between the phases. Video S1 shows respiratory phases when the cardiac phase is fixed to end‐diastole and cardiac phases when the respiratory phase is fixed to end‐exhalation, where the intermediate motion states can be appreciated. There was no motion from the fixed dimension, that is, no respiratory movement along the cardiac dimension and vice versa. Also, there was no noticeable motion blur, demonstrating that HAPTIC navigators are reliable for retrospective gating.

Finally, the performance of HAPTIC is tested on four challenging arrhythmia cases. Videos [Link], [Link] show real‐time movies of a 10‐second section from the 90‐second real‐time acquisition of each arrhythmia patient, chosen to demonstrate the performance of HAPTIC during irregular beats. Time plots show cardiac and respiratory PT and the ECG waveform for reference. The real‐time movies are synchronized to the waveforms for all four arrhythmia patients, with yellow highlighted regions showing successfully detected irregular beats and red highlighted regions where cardiac PT failed to detect irregular beats.

Table 1 shows the false positive/negative rates, mean delay, and jitter values for the arrhythmia patients. In patients 1 and 2, HAPTIC provided excellent false positive and negative rates (<1%) with acceptable jitter (<30 ms). However, in patients 3 and 4, we observed high failure rates (17.5% and 9.8%) and high jitter (>50 ms). It is worth noting that patient 4 had a moderate pleural effusion at the time of imaging that experienced signal fluctuations synchronous with the cardiac cycle. We speculate that the PT cardiac waveform was impacted by this motion, which may explain the abnormally high mean delay and poor triggering quality.

**TABLE 1:** High‐amplitude pilot tone with interference cancellation (HAPTIC) performance for each arrhythmia patient.

Patient no.	Mean delay (ms)	Jitter (ms)	No. of triggers	False positives	False negatives
1	122.7	9.2	92	0	0
2	130.9	24.5	105	0	1
3	146.6	56.9	80	12	2
4	490.8	84.8	112	0	11

*Note*: Failure rates (100× false triggers/total triggers) for the patients were 0%, 1%, 17.5%, and 9.8%, respectively. Videos [Link], [Link] show HAPTIC cardiac waveforms, ECG waveforms and HAPTIC respiratory waveforms and time‐synchronized movies for each volunteer. Note that patient 4 has a high delay, We believe this to be due to motion of the fluid in the pleural effusion, which can be appreciated from real‐time imaging (Video S5).

## 4 DISCUSSION

We demonstrate that HAPTIC is effective at 0.55T for both cardiac and respiratory gating. We demonstrate that EDITER can significantly reduce the residual high‐amplitude PT artifacts. The additional requirement of sniffer coils adds to system complexity, however, additional channels and coils are often readily available, such as the distant table‐integrated spine array elements used in this study. It is possible that the future shielding‐free scanners will also have sniffer coils built‐in.^20^

We have used a dipole antenna away from the bore to transmit the PT signal. A typical PT transmitter uses a non‐resonant loop coil in close vicinity to the chest. A comparison of two modes of operation is outside the scope of this work; however, there are practical advantages to the method used in this study. Having the PT transmitter separate from the receiver coils provides more flexibility in the imaging coil selection, not limiting to use an imaging coil with an integrated PT transmitter. Furthermore, the transmitter can be deployed independent of the scanner, simplifying the experiment setup, since the relative positioning of the transmitter will not depend on the subject body habitus or the MRI scan operator.

EDITER assumes no change in the system within a predetermined duration, thus will be less effective in removing artifacts related to changing PT signals. The extracted per‐coil motion information can be incorporated with EDITER to improve the interference mitigation further. Improvements in interference cancellation can reduce the noise increase induced by PT, which results in a more favorable jitter trade‐off.

In this work, we demonstrated HAPTIC's performance for retrospective gating. Specifically, the band‐pass filters were applied after the Fourier transform of the entire 90‐second waveform was taken. Similarly, PCA for navigator extraction is performed over the whole time waveform, making these operations non‐causal. To demonstrate prospective gating, extraction methods should be replaced by real‐time methods.^21^, ^22^ Alternatively, a vendor integrated PT device on a commercial 0.55T scanner may be used to demonstrate HAPTIC with prospective gating. The requirements for prospective gating, specifically the need for real‐time filters, may result in increased jitter, and may necessitate a higher PT voltage to achieve low jitter.

Patient 4 had an incidental finding of moderate pleural effusion on the MRI, and 800 cc of fluid was subsequently drained via thoracentesis. A significant cardiac PT signal was observed with a large delay with respect to the R‐wave. We believe this to be due to motion of the fluid in the pleural effusion, which can be appreciated from real‐time imaging. This is an interesting confounding factor for PT broadly, and warrants further investigation.

Finally, this work demonstrates initial feasibility of HAPTIC in a small cohort. An appropriate next step is to evaluate HAPTIC more comprehensively in a diverse patient cohort that includes a broad range of heart‐rate variability, body habitus, and physiological conditions, including disease states, that could impact PT performance.

## 5 CONCLUSIONS

HAPTIC enables reliable PT cardiac motion extraction at 0.55T, which was not possible before. The arising image artifacts due to increased PT amplitude are mitigated by the EDITER technique, an interference cancellation method, using three additional sniffer coils. HAPTIC is shown to be feasible and effective at 0.55T for both cardiac and respiratory gating, including the challenging case of cardiac arrhythmia.

## Supporting information


**Video S1.** Cardiac and respiratory phases from constrained reconstruction using pilot tone (PT) as the sole navigator. The video shows respiratory motion (left) during diastole and cardiac motion (right) during end‐exhalation.

**Video S2.** Arrhythmia patient 1, real‐time temporal finite difference (TFD) reconstructed movies, synchronized respiratory/cardiac pilot tone (PT) waveforms, and the reference electrocardiogram (ECG) waveform. The yellow highlighted region shows successfully detected irregular beats by cardiac pilot tone (PT) waveform.

**Video S3.** Arrhythmia patient 2, real‐time temporal finite difference (TFD) reconstructed movies, synchronized respiratory/cardiac pilot tone (PT) waveforms, and the reference electrocardiogram (ECG) waveform. The yellow highlighted region shows successfully detected irregular beats by cardiac PT waveform.

**Video S4.** Arrhythmia patient 3, real‐time temporal finite difference (TFD) reconstructed movies, synchronized respiratory/cardiac pilot tone (PT) waveforms, and the reference electrocardiogram (ECG) waveform. The yellow highlighted region shows successfully detected irregular beats by cardiac PT waveform. The red highlighted region shows where cardiac PT failed to produce detectable peaks for triggering. Note the irregular breathing pattern, which may be a factor in the failed PT navigator extraction.

**Video S5.** Arrhythmia patient 4, real‐time temporal finite difference (TFD) reconstructed movies, synchronized respiratory/cardiac pilot tone (PT) waveforms and the reference electrocardiogram (ECG) waveform. The red highlighted region shows where cardiac PT failed to produce detectable peaks for triggering. Note that the fluid in pleural effusion shows as a bright signal, as pointed by a red arrow. High delay with respect to the ECG R‐wave may be due to this fluid.

## Data Availability

Source code for the real‐time spiral sequence is shared at github.com/usc‐mrel/rtspiral_pypulseq/releases/tag/haptic‐mrm. Source code for pilot‐tone extraction and processing are shared at github.com/usc‐mrel/PylotToneMRI/tree/mrm‐submission. Source code for image reconstruction is shared at github.com/usc‐mrel/python‐ismrmrd‐server/releases/tag/haptic‐mrm. Sample raw data is shared at https://doi.org/10.5281/zenodo.14884661.
